# The Global Research Neglect of Unassisted Smoking Cessation: Causes and Consequences

**DOI:** 10.1371/journal.pmed.1000216

**Published:** 2010-02-09

**Authors:** Simon Chapman, Ross MacKenzie

**Affiliations:** School of Public Health, University of Sydney, Australia

## Abstract

Simon Chapman and Ross MacKenzie review the evidence and argue that health promotion messages should emphasize that the most successful method used by most ex-smokers is unassisted cessation.

**Summary Points**

- Research shows that two-thirds to three-quarters of ex-smokers stop unaided. In contrast, the increasing medicalisation of smoking cessation implies that cessation need be pharmacologically or professionally mediated.
- Most published papers of smoking cessation interventions are studies or reviews of assisted cessation; very few describe the cessation impact of policies or campaigns in which cessation is not assisted at the individual level.
- Many assisted cessation studies, but few if any unassisted cessation studies, are funded by pharmaceutical companies manufacturing cessation products.
- Health authorities should emphasise the positive message that the most successful method used by most ex-smokers is unassisted cessation.

## Introduction

As with problem drinking, gambling, and narcotics use [1]–[9] population studies show consistently that a large majority of smokers who permanently stop smoking do so without any form of assistance [10]–[15]. In 2003, some 20 years after the introduction of cessation pharmacotherapies, smokers trying to stop unaided in the past year were twice as numerous as those using pharmacotherapies and only 8.8% of US quit attempters used a behavioural treatment [16]. Moreover, despite the pharmaceutical industry's efforts to promote pharmacologically mediated cessation and numerous clinical trials demonstrating the efficacy of pharmacotherapy, the most common method used by most people who have successfully stopped smoking remains unassisted cessation (cold turkey or reducing before quitting [16],[17]). In 1986, the American Cancer Society reported that: “Over 90% of the estimated 37 million people who have stopped smoking in this country since the Surgeon General's first report linking smoking to cancer have done so unaided.” [18]. Today, unassisted cessation continues to lead the next most successful method (nicotine replacement therapy [NRT]) by a wide margin [15],[16].

Yet, paradoxically, the tobacco control community treats this information as if it was somehow irresponsible or subversive and ignores the potential policy implications of studying self-quitters. Unassisted cessation is seldom emphasised in advice to smokers [19]. We know of no campaigns that highlight the fact that most ex-smokers quit unaided even though hundreds of millions have done just that. Reviews typically give unassisted cessation cursory attention [20], framing it as a challenge to be eroded by persuading more smokers to use pharmacotherapies: “Unfortunately, most smokers …fail to use evidence-based treatments to support their quit attempts” [21]; “If there is a major failing in the UK approach, it is not that it has medicalised smoking, but that it has not done so enough.” [22]. Clinical guidelines also ignore unassisted cessation [8]. Finally, although the US National Center for Health Statistics routinely included a question on “cold turkey” cessation in its surveys between 1983 and 2000, this question disappeared in 2005 [23].

Because of these prevalent attitudes, smoking cessation is becoming increasingly pathologised, a development that risks distortion of public awareness of how most smokers quit to the obvious benefit of pharmaceutical companies. Furthermore, the cessation research literature is preoccupied with the difficulty of stopping. Notably, however, in the rare literature that has bothered to ask [24], many ex-smokers recall stopping as less traumatic than anticipated. For example, in a large British study of ex-smokers in the 1980s, before the advent of pharmacotherapy, 53% of the ex-smokers said that it was “not at all difficult” to stop, 27% said it was “fairly difficult”, and the remainder found it very difficult [25].

We recently hypothesized that research into smoking cessation follows what we call “the inverse impact law of smoking cessation.” This law posits that “the volume of research and effort devoted to professionally and pharmacologically mediated cessation is in inverse proportion to that examining how most ex-smokers actually quit. Research on cessation is dominated by ever-finely tuned accounts of how smokers can be encouraged to do anything but go it alone when trying to quit—exactly opposite of how a very large majority of ex-smokers succeeded.” [26]

In this Policy Forum, we test this law and, because a recent review of Cochrane selected randomized controlled trials of NRT [27],[28] found that while 51% of industry-funded trials reported significant cessation effects, only 22% of nonindustry trials did, we also test the hypotheses that research on pharmaceutically mediated cessation is frequently conducted by researchers supported by pharmaceutical companies and that support for research into unassisted cessation and nonpharmaceutical interventions is less common. Throughout this Policy Forum, by assisted cessation, we mean any pharmacotherapy or any individual or group behavioural or cognitive intervention. By unassisted cessation, we mean approaches that involve none of these interventions but instead include interventions such as changes in tobacco tax, smoking restrictions, or public awareness campaigns designed to stimulate cessation. We then consider why research into how most people stop smoking is being neglected and reflect on the potential negative consequences for public health of repeatedly megaphoning the message that “serious” cessation is assisted cessation, a message that implies that unassisted cessation is less worthy of research attention, publicity, and consideration by quitters. Finally, we suggest how the message that smokers are getting about cessation should be adjusted to help more people quit.

## Testing the Inverse Impact Law of Smoking Cessation

On May 12, 2009, we searched Medline for “smoking cessation,” limiting results to English language original articles, meta-analyses, and reviews published in 2007 and 2008. Of the 885 papers returned, we excluded those that dealt specifically with the effects of cessation on behavioural, cognitive or affective variables, study recruitment research, health economics, and those papers that had a different primary focus, such as smoking-related diseases.

**Figure 1:**
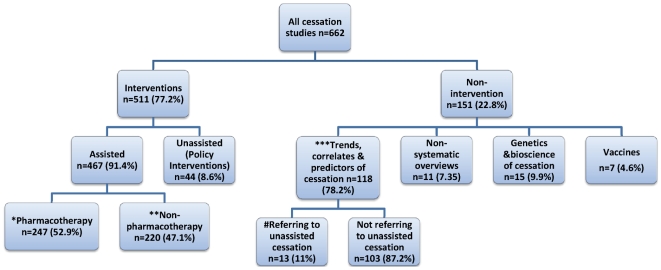
Focus of original research and reviews of 662 smoking cessation papers indexed by Medline, 2007–2008 (percent of all papers). *, Pharmacotherapy: NRT only 57; bupropion only 19; varenicline only 26; combination/head-to-head trials 56; other pharmacotherapy 20; pharmacotherapy versus nonpharmacotherapy 7; pharmacotherapy with counselling 49; meta-analyses/systematic reviews 8; reduced nicotine cigarettes 2; smokeless tobacco 3. **, Nonpharmacotherapy: community cessation centres, groups, counselling 28; primary health care 29; hospital-based or referrals 39; workplace programs 6; schools/youth 16; quitlines 26; phone or posted initiated by professionals 16; Web-based 32; combination quitline/Web/calls 9; pamphlets/books 2; spirometry as motivator 2; acupuncture/acupressure 3; exercise 6; meta-analyses/systematic reviews 6. ***, Trends, correlates and predictors. Whole or special populations 82; youth 16; primary health care 6; hospital patients 12; workplaces 2. #, Three articles were unobtainable.

Of the 662 papers that met our inclusion criteria, 511 were studies of cessation interventions. The other 118 were mainly studies of the prevalence of smoking cessation in whole or special populations. Of the intervention papers, 467 (91.4%) reported the effects of assisted cessation and 44 (8.6%) described the impact of unassisted cessation (Figure 1). Some of those quitting as a consequence of unassisted cessation policies or programs would have used assisted methods, but these papers reported only on smoking status, not on *how* those who quit did so. Of the studies describing assisted interventions, 247 (52.9%) involved pharmacotherapy and 220 (47.1%) nondrug interventions. Of the papers describing cessation trends, correlates, and predictors in populations, only 13 (11%) contained any data on unassisted cessation.

We then randomly chose 30 papers that considered assisted cessation interventions, 30 that considered unassisted cessation interventions, and 30 that discussed the prevalence of smoking cessation to test the hypothesis these groups of papers would not differ in terms of whether authors and/or studies had received support from a pharmaceutical company manufacturing smoking cessation products. For papers that contained no declarations of competing interests and/or pharmaceutical industry funding, we emailed the corresponding authors to request this information. Where no replies were received, we examined these authors' previous publications on cessation from the past 5 years for such declarations.

Of the 84 papers for which competing interest information was available, 12/25 (48%) of pharmacotherapy intervention studies, 3/29 (10.3%) of nonpharmacotherapy intervention studies, and 0/30 of unassisted cessation studies had at least one author declaring support from a company manufacturing cessation products and/or research funding from such a company (*p*<0.001). Five of the six authors who did not respond to requests for information on competing interests were previously involved in studies on pharmacological interventions for cessation.

## Why Does the Research Concentrate on Assisted Cessation?

With approximately two-thirds [16] to three-quarters [15] of ex-smokers stopping unaided, our finding that 91.3% of recent intervention studies focused on assisted cessation provides support for the inverse impact law of smoking cessation [26], although further studies are needed to confirm that the bias towards studies on assisted cessation interventions that we discovered is a long-standing one and not peculiar to the years we studied. We believe there are three main synergistic drivers of the research concentration on assisted cessation and its corollary, the neglect of research on the natural history of unassisted smoking cessation. These are: the dominance of interventionism in health science research; the increasing medicalisation and commodification of cessation; and the persistent, erroneous appeal of the “hardening” hypothesis.

### The Dominance of Interventionism

Most tobacco control research is undertaken by individuals trained in positivist scientific traditions. Hierarchies of evidence give experimental evidence more importance than observational evidence [29],[30]; meta-analyses of randomized controlled trials are given the most weight. Cessation studies that focus on discrete proximal variables such as specific cessation interventions provide “harder” causal evidence than those that focus on distal, complex, and interactive influences that coalesce across a smoker's lifetime to end in cessation. Specific cessation interventions are also more easily studied than the dynamics and determinants of cessation in populations [31]. Experimental research focused on proximal relationships between specific interventions and cessation poses fewer confounding problems and sits more easily within the professional norms of scientific grant assessment environments, which are populated largely by scientists working within the positivist tradition.

The dominance of the experimental research paradigm is amplified by pharmaceutical industry support for drug trials. More than half the papers we found on assisted cessation were pharmaceutical studies and, unsurprisingly, these were much more likely than papers on nonpharmacological interventions to have industry-supported authors. Companies have an obvious interest in research about the use and efficacy of their products and less interest in supporting research into forms of cessation that compete with pharmacotherapy for the cessation market.

The availability of pharmaceutical industry research funding—often provided without the lengthy processes of open tender or independent peer review—can be highly attractive to researchers. Furthermore, it is often observed that “research follows the money,” with scientists being drawn to well-funded research areas [32]. The large pool of research funding for pharmacotherapeutic cessation may cause researchers to gravitate toward such studies while those interested in the natural history of smoking cessation have to secure funding through highly competitive public grant schemes.

This greater availability of funding for certain sorts of research produces a distorted research emphasis on pharmacotherapy that, when combined with the industry's formidable public relations abilities and direct-to-consumer advertising, concentrates both scientific and public discourse on cessation around assisted pharmacotherapy. In 2006, the global NRT market was estimated at $1.7 billion [33]. The pharmaceutical industry places more messages about quitting in front of smokers than any other source: in the USA, there are 10.37 pharmaceutical cessation advertisements per month but only 3.25 government and NGO cessation messages [34].

### The Medicalisation and Commodification of Cessation

Tobacco use, like other substance use, has become increasingly pathologised as a treatable condition as knowledge about the neurobiology, genetics, and pharmacology of addiction develops. Meanwhile, the massive decline in smoking that occurred before the advent of cessation treatment is often forgotten. Warner [35] documented this decline, which started following news coverage of the 1964 report of the US Surgeon General. He noted that “per capita consumption likely would have exceeded its actual 1975 value by 20 to 30 per cent” without this decline. Other than the first small pack warnings that appeared from 1966 in the USA, this effect occurred without any elements of today's comprehensive approaches to tobacco control.

In 1975, Renaud wrote of the fundamental tendency of capitalism to “transform health needs into commodities … When the state intervenes to cope with some health-related problems, it is bound to act so as to further commodify health needs.” [36]. The burgeoning commodification of cessation by manufacturers of both effective and ineffective [37] drugs seems to have induced a kind of professional amnesia in tobacco control circles about the millions who quit in the decades before the dominance of the contemporary smoking cessation discourse by pharmacotherapy. As Granfield and Cloud remarked about the substance abuse field's aversion to studying unassisted recovery by narcotics addicts, the dominance of assisted cessation in the tobacco control field “has a common tendency to exclude the experiences of people who do not fit into prevailing models of substance problems and treatment” [2].

### The Persistent, Seductive, and Erroneous Appeal of the “Hardening” Hypothesis

This hypothesis predicts that where “smoking prevalence is lowest or the most progress in reducing smoking prevalence has been made, the remaining smokers are more likely to be ‘hard-core’, or refractory to a policy and/or treatment interventions, because the people who have quit were less dependent on nicotine, and/or more motivated to quit.” [38]. The intuitive attractions of this hypothesis generated an entire US National Cancer Institute monograph [39]. Hardening adherents argue that ex-smokers are dominated by those who were not heavily addicted and so who were better able to quit unaided and that a greater proportion of today's smokers, said to be more addicted, cannot succeed alone and need help. This hypothesis has been heavily criticised [40]. Most recently, data on smoking in 50 US states for 2006–2007 indicate that the mean number of cigarettes smoked daily, the percentage of cigarette smokers who smoke within 30 minutes of waking, and the percentage who smoke daily are all significantly lower in US states with low smoking prevalence, compelling evidence against the hardening hypothesis [38].

## Does Research into Assisted Cessation Apply to the Real World?

Accumulated evidence from clinical trials shows unequivocally that those who use NRT in trials have 50%–70% greater success than those using placebo [28]. But clinical trial conditions typically overstate real world effectiveness because of factors such as trial participants getting free drugs and “Hawthorne” effects caused by the research attention paid to participants [41] and the participants' desire to please the researchers with whom they interact. Moreover, Mooney et al. [42] found that only 23% of NRT placebo-controlled trials assessed blindness integrity and 71% of these trials found that the participants could detect if they had been assigned to the active agent, a rate significantly above chance.

The results from a smaller, but growing, literature examining “real world” use provides a more sobering assessment of the potential of this intervention to significantly improve population rates of cessation. Walsh's review concluded that it is “not yet established that NRT alone is superior to self-quitting in an unsupported OTC [over the counter] environment” [41] and noted major limitations in Hughes' earlier, more optimistic meta-analysis [43].

For the clinical trial efficacy of NRT to be replicated in the real world, smokers may need to have some form of support during their cessation efforts but few smokers are interested in engaging with smoking cessation support services. In Australia, for example, in spite of the national quitline number appearing on every cigarette pack and in every government quit message, only 3.6% of smokers called the quitline in a year [44]. In 2000–2004, in the UK area with the highest reported cessation support participation rate, only 6% of smokers used the available support services [45]. Prospects for engaging larger proportions of smokers in more intensive interventions seem poor.

Overall, population level analyses of the impact of the proliferation, deregulation, and widespread promotion of NRT and other pharmacotherapies have failed to show any significant, sustained impact on smoking prevalence, despite the conclusions of clinical trials. Cummings and Hyland's 2005 review concluded that: “Time series analyses of national cigarette consumption and NRT sales from 1976 to 1998 suggest that sales of NRT were associated with a modest decrease in cigarette consumption immediately following the introduction of the prescription nicotine patch in 1992. However, no statistically significant effect was observed after 1996, when the patch and gum became available OTC. … annual quit rates as well as age-specific quit ratios remained stable” [46]. Similar conclusions were reached for Massachusetts [47] and California [48]. Most recently, Wakefield et al. assessed the impact of televised antismoking advertising, cigarette price, sales of NRT and bupropion (a smoking cessation drug), and NRT advertising by examining monthly Australian smoking prevalence from 1995 to 2006. They found that, unlike antismoking advertising and price, neither NRT or bupropion sales nor NRT advertising had any detectable impact on smoking prevalence [49]. Although this lack of effect may have been due to power limitations (some 40% of smokers make an attempt to quit each year, a fraction of these use pharmaceutical aids, and an even smaller fraction quit, which means that extremely large population samples are needed to detect any effect of these interventions), it hardly inspires confidence that assisted cessation makes a major contribution to reducing smoking in populations.

The public is often advised that assistance at least doubles cessation rates. But while the clinical trial literature consistently shows higher quit rates from assisted than unassisted cessation, population studies show the opposite. For example, a 1990 US study found 47.5% of those who tried to quit unaided over 10 years were successful, compared with 23.6% using cessation programs [10]. Schachter noted that treatment-aided cessation rates may be lower than unassisted quit rates because of selection bias: those seeking treatment are likely to have made unsuccessful quit attempts and may be more failure-prone [50]. In 2008, Shiffman et al. reiterated this point: “Further, smokers self-select for treatment, based on their perceived need and expectations of difficulty quitting …so treatment-seeking itself can index risk for failure, undermining the validity of comparisons of outcome between treatment-seekers and non-seekers.” [16],[51].

A final example of how promoters of assisted cessation can maintain their position in the face of apparently contradictory results comes from a recent US study of unplanned cessation [52], which corroborated previous findings [53],[54] by reporting that unplanned cessation attempts were twice as successful as planned attempts and, significantly, that most unplanned quit attempters did not use any assistance. The authors noted that: “Given the evidence that use of medication can double success rates, it is surprising that even without this assistance unplanned quitters were more likely to be successful. It seems important to find ways to combine the favorable prognosis of unplanned quit attempts with the benefit of medication, for example, by ensuring easy, rapid access to medication.” They then suggested the removal of barriers to NRT sale such as prescription-only or pharmacy-only status, failing to note that these barriers had already been removed in the USA. The “surprise” expressed by the authors of this paper (all of whom had declared support from the pharmaceutical industry) seems revelatory of the myopic hold that assisted smoking cessation can have on the population-wide picture of how people quit.

## The Consequences of the Research Neglect of Unassisted Cessation

There has been a long history of criticism of the medicalisation of everyday life [55] to service social control [56] and medical and pharmaceutical industry profits [57]. As Caron et al. note: “the classic drawback of medicalization is its reductionism, which places excessive emphasis on the biological and individual determinants of disease at the expense of a more holistic perspective that emphasizes the social, cultural, and environmental contributions to disease and illness.” [58]. The neurobiology of nicotine dependency is well-established [59], and understanding of its genetics [60] is accelerating. But plainly, with the existence of many millions of unassisted ex-smokers and given the ways that international variations in their distribution reflect social, cultural, and public-health policy variables, smoking cessation in populations is explained by far more than neurobiology and pharmacology.

The persistent messaging that nicotine addiction is refractory and stopping unaided will be futile deflects attention away from what is by far the most common story of cessation: people doing it without professional or therapeutic help. When citizens have common, self-limiting ailments and traits and behaviours are regularly redefined as needing treatment, avoidable iatrogenic consequences and burgeoning health care expenditure can follow. But the steady erosion of human agency as populations lose confidence in their own ability to change unhealthy practices is perhaps of greater concern. Several negative consequences arise from smokers being increasingly imbued with the message that serious efforts at cessation require treatment.

It is understandable that smokers might feel it would be foolish to attempt to stop unaided when unassisted cessation is dismissed in pharmaceutical industry–supported demonstrably misleading propaganda [61] by statements such as: “It is hopelessly outdated to suggest: ‘willpower alone is enough to quit’. … Quitting ‘cold turkey’ does not generally translate into sustained abstinence from tobacco, and results in unnecessarily low rates of success for most smokers.” [62]; and: “[the] narrow ‘de-medicalized’ view of nicotine addiction …[has] conceivably perpetuated the epidemic [and] contributed to innumerable deaths” [62]. Because most assisted cessation attempts end in relapse, such “failure” risks are interpreted by smokers as “I tried and failed using a method that my doctor said had the best success rate. Trying to quit unaided – which I never hear recommended – would be therefore sheer folly.” Such reasoning might well disempower smokers and inhibit quit attempts through anticipatory, self-defeating fatalism [63].

## Why Study Unassisted Cessation?

In any endeavour, whether it be health-related such as weight loss, physical activity or ending narcotics use, or wider achievements such as business success or artistic virtuosity, it would seem reasonable to consider that studying those who had succeeded or excelled might reveal factors that might be valuable to others. Studying the habits, attitudes, routines, and environments of people who succeed where many others fail is commonplace in other fields so why not study unassisted smoking cessation?

The relatively few studies reporting on people who have quit unaided provide important information about factors associated with motivating quit attempts and with successful unaided cessation. Some of these factors are amenable to change via legislation or mass-reach public-awareness campaigns. Smoke-free homes [15] and workplaces [64], family and social support [65], bold pack warnings [66], price, and hard-hitting, well-funded campaigns [49] have all been associated with increased cessation activity and success, and relapse has been associated with exposure to social smoking cues [67].

Warner and Mackay argue that: “We can have our cake and eat it too” [68], stating that further resources and emphasis should be given to treating tobacco dependence *as well as* to public-health, population-focused approaches to promoting cessation. Certainly, smoking cessation treatment is one of the most cost-effective interventions in modern medicine [69], and wealthy nations can afford both approaches. However, today's largest tobacco markets are nations with massive populations on low incomes for whom pharmacotherapy is prohibitively expensive. In Indonesia for example, 3 months of NRT costs as much as 7 year's supply of cigarettes, placing NRT totally out of the reach of all but the wealthy [70]. NRT would thus seem to be largely irrelevant to population-wide cessation goals in many low- and middle-income nations.

Such nations emphatically cannot afford “both” and are often still struggling to fund basic primary health care, public-health, and sanitation infrastructures. Population-oriented, mass-reach tobacco control policy and programs are the exceptions in such nations. In our view, it would be a disaster for tobacco control progress if such nations were to be influenced to proliferate labour-intensive UK-style [71] models of assisted cessation before they implemented comprehensive and sustained population-focused cessation policies and programs. In most nations, tobacco control is in its nascent phase. Siphoning resources and scarce personnel into smoking cessation strategies that reach relatively few and help even fewer would be grossly inequitable.

## What Message Should Smokers Get about Cessation?

The persistence of unassisted cessation as the most common way that most smokers have succeeded in quitting is an unequivocally positive message that, far from being suppressed or ignored, should be openly embraced by primary health care workers and public-health authorities as the front-line, primary “how” message in all clinical encounters and public communication about cessation. Put another way, a failure to emphasise that most smokers have always stopped unaided would be like claiming that most domestic cooks attend cooking classes. Along with motivational “why” messages designed to stimulate cessation attempts, smokers should be repeatedly told that cold turkey and reducing-then-quitting are the methods most commonly used by successful ex-smokers, that more smokers find it unexpectedly easy or moderately difficult than find it very difficult to quit [25], that many successful ex-smokers do not plan their quitting in advance [52]–[54], and that “failures” are a normal part of the natural history of cessation—rehearsals for eventual success. Lessons learned from researching policy tractable, social support, and personal behavioural (“quit tips”) variables associated with successful cessation should be fed into policy and program planning. Talk of unassisted cessation being “the enemy” of evidence-based cessation should be roundly criticised as both incorrect and unhelpful. Unfortunately, the ability of manufacturers to promote their products through advertising is likely to “drown out” the perspective we urge. We suggest, therefore, that public sector communicators should be encouraged to redress the overwhelming dominance of assisted cessation in public awareness, so that some balance can be restored in smokers' minds regarding the contribution that assisted and unassisted smoking cessation approaches can make to helping them quit smoking.

**What Message Should Smokers Get about Cessation?**

- There is good news about cessation: in a growing number of countries, there are more ex-smokers than smokers.
- Up to three-quarters of ex-smokers have quit without assistance (“cold turkey” or cut down then quit), and unaided cessation is by far the most common method used by most successful ex-smokers.
- A serious attempt at stopping need not involve using NRT or other drugs or getting professional support.
- Early “failure” is a normal part of trying to stop. Many initial efforts are not serious attempts.
- NRT, other prescribed pharmaceuticals, and professional counselling or support also help many smokers, but are certainly not necessary for quitting.

## Abbreviations

NRT: nicotine replacement therapy
